# Independent Component Analysis for Brain fMRI Does Indeed Select for Maximal Independence

**DOI:** 10.1371/journal.pone.0073309

**Published:** 2013-08-29

**Authors:** Vince D. Calhoun, Vamsi K. Potluru, Ronald Phlypo, Rogers F. Silva, Barak A. Pearlmutter, Arvind Caprihan, Sergey M. Plis, Tülay Adalı

**Affiliations:** 1 Medical Image Analysis Lab, The Mind Research Network, Albuquerque, New Mexico, United States of America; 2 Department of Electrical and Computer Engineering, University of New Mexico, Albuquerque, New Mexico, United States of America; 3 Department of Computer Science, University of New Mexico, Albuquerque, New Mexico, United States of America; 4 Hamilton Institute and Department of Computer Science, National University of Ireland Maynooth, Co. Kildare, Ireland; 5 Department of Computer Science and Electrical Engineering, University of Maryland Baltimore County, Baltimore, Maryland, United States of America; National Research & Technology Council, Argentina

## Abstract

A recent paper by Daubechies et al. claims that two independent component analysis (ICA) algorithms, Infomax and FastICA, which are widely used for functional magnetic resonance imaging (fMRI) analysis, select for *sparsity rather than independence*. The argument was supported by a series of experiments on synthetic data. We show that these experiments fall short of proving this claim and that the ICA algorithms are indeed doing what they are designed to do: identify *maximally independent sources*.

## Introduction

Independent component analysis (ICA) [1]–[4] is a widely used signal processing approach that has been applied to areas including speech separation, communications, and functional magnetic resonance (fMRI) data analysis. Given a set of linearly mixed observations, recovering the underlying components is an ill-defined problem. However, the assumption of independence among the sources turns out to be surprisingly powerful and effective for a wide range of problems in various practical domains.

Sparsity is another commonly imposed assumption that arises naturally from the principle of parsimony: the simplest explanation is preferred. Sparsity is also motivated by evidence of neuronal coding efficiency and sparse coding in the nervous system. Sparse representations can help avoid the problem of overfitting while also leading to solutions that are easier to interpret. Applications of sparse signal processing methods include dictionary learning [5], speech separation [6], and feature learning [7].

Daubechies et al. [8] claims that ICA for fMRI optimizes for sparsity rather than independence. This is established by first noting that Infomax and FastICA are two algorithms widely used for fMRI analysis and then showing that they separate sparse components better than independent ones on a synthetic dataset. Recreating the synthetic dataset and conducting additional experiments shows that the FastICA and Infomax algorithms indeed do what they are designed to do. Both ICA algorithms can separate sources with either high or low degrees of sparsity, as long as the distributional assumptions of the algorithms are approximately met. To understand the conditions under which these algorithms work requires correct interpretation of what the sources are in an ICA formulation. We examine *exactly what* the sources are in the examples given in Daubechies et al. [8] and show that there is an important mismatch between the concept of source therein and what an ICA source actually is, which is ultimately at the heart of the unsupported conclusions presented in Daubechies et al. [8].

## Review and Critique of the Presented Evidence

We now briefly review the evidence presented in Daubechies et al. [8] to support the claim that Infomax [3] and FastICA [9] select for sparsity and not independence. Following Daubechies et al., we refer to the versions of the two algorithms with their default nonlinearities, sigmoid for Infomax, which is a good match for sources with super-Gaussian distributions, and the high kurtosis nonlinearity for FastICA. Daubechies et al. [8] exhibits experimental results in which 1) ICA algorithm performance suffers when the assumptions on the sources are violated, and 2) ICA algorithms can separate sources in certain cases even if the sources are not strictly independent. The two points above, both of which were already widely known in the ICA community at the time, are not sufficient evidence to support the claim that ICA selects for sparsity and not independence. In addition, Daubechies et al. [8] presents a case in which the sources are somewhat dependent but also very sparse, and Infomax and FastICA do well. This result is used to claim that it is sparsity rather than independence that matters. We augment this experiment with new evidence which shows that the same ICA algorithms perform equally well in the case of both minimum and maximum sparsity (using the definition of sparsity in Daubechies et al. [8]), suggesting that the role of sparsity (if any) is minor in the separation performance.

Additional evidence in Daubechies et al. [8] involves a discussion of sparsity in which it is claimed that ICA can separate Gaussian sources (See Legend of Fig.8 in Daubechies et al. [8]) which are also sparse (utilizing a definition of sparsity different from the one initially provided in Daubechies et al. [8]). If true, such a result would support their claim about the role of sparsity in ICA, since it is well established that blind ICA algorithms are not able to separate two or more Gaussian sources. However, as we show, in that example the sources as they are generated are highly non-Gaussian, and the sparsity mentioned in Daubechies et al. [8] does not actually refer to the sources. Rather, it refers to vectors that span parts of both sources. This renders their statement incorrect and hence, does not support the claim being made (see Section “Sparsity and sources that are mixture of Gaussians” for details).

Finally, the paper [8] is focused on showing cases where FastICA and Infomax perform well or poorly, and from these cases the claim is made that this applies to ICA of fMRI in general. There is mention that a more general algorithm [10] does not work for fMRI, but there is no evidence presented to support this claim. As we later discuss in Section “On the application of ICA to fMRI,” other ICA algorithms had indeed been used on fMRI data with success, at the time of the publication [8]. Since then, more flexible ICA algorithms have been applied to fMRI data and noted to demonstrate even better performance than the widely used Infomax and FastICA [11]. Hence, while emphasizing that Infomax and FastICA are not the only two algorithms that have been applied to fMRI analysis, we also note that the prevalence of the use of these two is largely due to the availability of the code for these algorithms and their default use in toolbox implementations for fMRI analyses. Since most of the fMRI community does not specialize in the development of blind source separation algorithms, they have since opted in general for the use of these two implementations. And although they do perform reasonably well on fMRI data, sparsity is not the major driver of this success.

## Experiments on Synthetic Data: Boxes

We now describe the synthetic dataset used in the original paper [8]. Two components 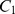 and 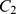 are generated as follows: 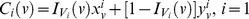, where the 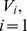, are different subsets of 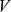, and 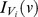 denotes the indicator function for 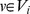; the variables 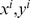 are independent random variables and 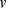 is the sample index. In Example 1 [8], the cumulative distribution functions (CDFs) of 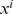 are identical and given by 
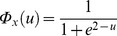
, i.e., logistic distributions with mean 2 and scale parameter 1 (the standard deviation is 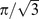). In Example 2 [8], 
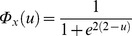
 (logistic with mean 2, scale parameter 0.5, and standard deviation 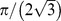). Here, 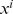 correspond to the activations. Similarly, the CDFs of 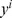 are identical and given by 
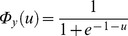
, i.e., logistic distributions with mean –1 and scale parameter 1 (the standard deviation is 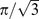), where 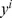 correspond to the background. The mixtures are given by: 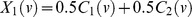 and 
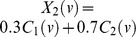
. We have 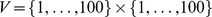, and in the case of “medium boxes”, 
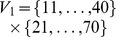
 and 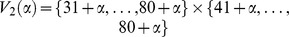, 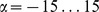. Furthermore, for Example 2 [8], in the case of “small boxes”, the sample support sets are 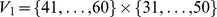 and 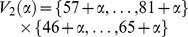, and in the case of “large boxes”, 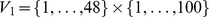 and 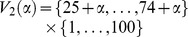, 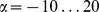. In all cases, 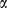 controls the relative position of the boxes, and 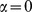 gives statistical independence between 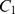 and 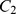.

## The Statistical Properties of Synthetic Data in Daubechies et al. [8]

Daubechies et al. [8] argues, based largely on results from synthetic datasets using boxes to represent activated regions of a component (see details above), that it is sparsity rather than independence that enables the recovery of the components. However, the case where the algorithms fail is actually due to a mismatch between the algorithms’ assumptions and the *statistical properties* of the simulated data. In addition, we demonstrate a case where they perform best, which corresponds to almost the *lowest* sparsity (i.e., not sparse). To facilitate cross-referencing, in the results presented herein, we use the first definition of sparsity (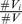) provided in Daubechies et al. [8]. Note, however, that the quantification of sparsity may be ambiguous: see Section “On the definition of sparsity” below, and the two definitions of sparsity in Daubechies et al. [8].

**Figure 1:**
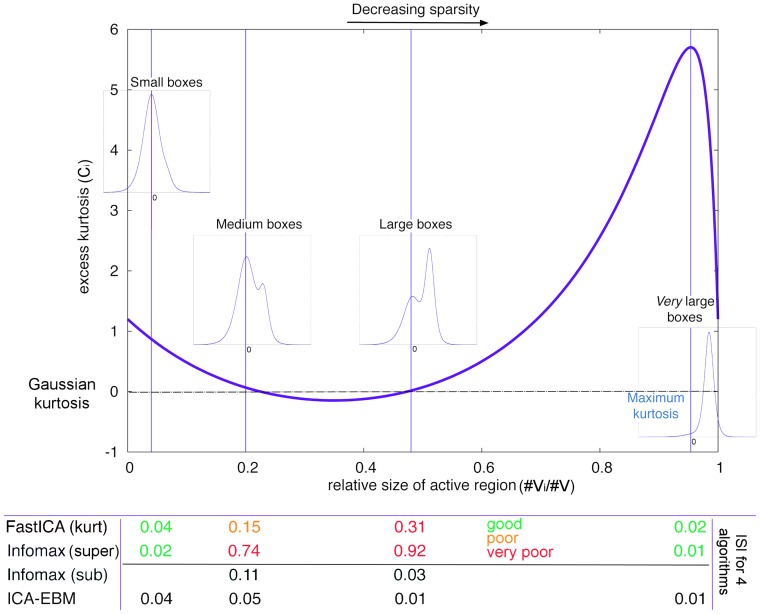
The excess kurtosis of a source 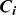 as a function of the relative size of the active region. A Gaussian has zero excess kurtosis. Here 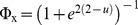 as in Example 2 of the original paper [8]. The four vertical lines at correspond to the relative sizes of the small box, the medium box, the large box, and a very large box corresponding to the maximal kurtosis case. Note that the medium and large box experiments have near zero excess kurtosis, *i.e., kurtosis value matching that of a Gaussian*. In addition, the pdfs of these sources are bimodal (see inset figures), ensuring that ICA algorithms designed for unimodal super-Gaussian distributions such as Infomax and FastICA with standard parameter settings, will likely fail. At the bottom of the figure are the ISI values (see Equation (2)) for the various algorithms at those four points (see Table 1 for full list). Also note the best separation performance of Infomax and FastICA for the maximum kurtosis case, which corresponds to almost the *lowest* level of sparsity.

Let us first concentrate on the choice of sources. In Figure 1, we see the excess kurtosis of the simulated sources changes with the relative size of the activation region. For medium and large boxes, the two cases where Infomax and FastICA are noted to fail, the kurtosis values are close to that of a Gaussian (i.e., zero), almost corresponding to the two zero-crossings. Moreover, in these cases the distributions are bimodal, far from the unimodal super-Gaussian assumptions that underpin the nonlinearities of Infomax and FastICA used in Daubechies et al. [8]. The paper [8] showed that Infomax with a non-linearity matched to super-Gaussian sources fails for medium and large boxes, roughly regardless of the relative position of the box; but it was *not* noted that the sources 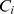 were very close to Gaussian (in the sense of kurtosis) and in disagreement with the nonlinearity. Both of these facts create very challenging scenarios for ICA algorithms based on the assumption of unimodal, super-Gaussian sources, as is the case in Infomax and FastICA, and of course sources are not even close to the “ideal” setup for these algorithms, contrary to the claim on p.10418 in Daubechies et al. [8]. In fact, under these scenarios components would *not* be expected to be well separated with either of these algorithms – because of the mismatch of the distribution (for Infomax) and an approximately zero kurtosis (for FastICA).

It is noted in Daubechies et al. [8] that the sources are designed by matching their cumulative distribution function (CDF) to the nonlinearity of the algorithm, resulting in “optimal” detectability for Example 1 [8], and (intentionally) enforcing a “slight mismatch” for Example 2 [8]. First, these two CDFs are actually the same, except for a scaling factor, which would translate to the so-called scaling ambiguity in ICA. More importantly though, there is a mismatch in vocabulary between what is being identified as the underlying ICA source in Daubechies et al. [8] and *what it actually is* in the experiment. Specifically, the nonlinearity matches solely to the activation part of the components thereby neglecting the background, whereas the ICA source is to be understood as a combination of the two, and thus has a distribution that is a *mixture distribution*, i.e., a weighted sum of both activation and background distributions. Hence the claim (p. 10418, 1st column): “For the first choice, the parameters of our ICA implementations provide optimal ‘detectability’ in the sense that the nonlinear function defined by the parameter setting of the algorithm coincides with the CDF of the signal source;” is incorrect since the *source* in this linear source separation framework cannot refer to only a part of the underlying distribution. As it turns out, in Example 2 [8] there is actually a *large* mismatch (rather than a “slight mismatch”) with respect to the algorithm’s nonlinearity in that the source distributions are essentially bimodal (see Figure 1, medium box inset).

## Boxes Revisited

In the boxes experiment, there are four quantities that are varied: the relative position of the boxes (controlling the amount of overlap), the size of the boxes (small, medium, large), the distribution of the marginal (i.e., the source 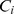), and the joint distribution. The shift of the box changes the amount of overlap and, thus, the joint distribution/dependence. The box size controls the sparsity (small box  =  high sparsity, large box  =  low sparsity) through the proportion of 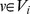, and thus changes the marginal distribution of the sources 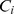. Clearly, there is dependence between all four quantities, which makes interpretation of the results ambiguous at the least. This is a side effect of the way the sources are sampled in Daubechies et al. [8], which is not independent and identically distributed (i.i.d.) due to the use of the indicator function to define boxes in the spatial map (the sampling distribution is not identical but, instead, conditioned on the location of each sample). With such a design it is very difficult to understand what causes the experimental differences, which is contrary to the claim [8] that it is “easy to change each of these characteristics separately”. In addition, in the experiments, a single fixed mixing matrix is used, which is not an ideal way to evaluate performance as results are then biased to a specific (and unjustified) set of mixing matrix parameter choice.

In order to furnish a clear, unbiased interpretation of the effect of the marginal source distributions (closely related to the box-sizes in Daubechies et al. [8]) on the performance of ICA algorithms that exploit non-Gaussianity, we first eliminate the effects of all other parameters by limiting ourselves to the case of two *independent* sources 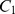 and 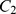. Then we generate samples directly from marginal distributions that match those in Daubechies et al. [8]. Since the sources defined in Daubechies et al. [8] have distributions that are of mixture type, we can write the CDF of each source 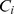 as 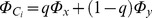, where 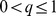 with 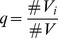, and then draw a set of i.i.d. samples. Under these conditions, the joint distribution of all samples reads: 
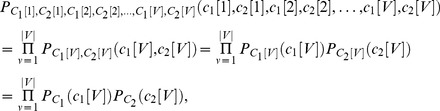
(1)


where 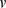 is the sample index and 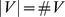. The first equality follows from independent sampling, the second equality from the independence between components 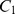 and 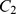, and the third equality from the samples being identically distributed (same distribution regardless of the sample index 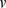). As such, we may generate all samples using independent samples from the inverse CDF transforms 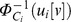, where 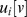, 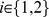, 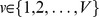 are i.i.d. samples from the independent random variables 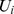, 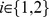, uniformly distributed on 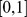, and 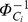 is the inverse CDF of the mixture distribution 
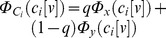
. Here 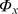 is the logistic distribution for activation and 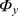 is the logistic distribution for background as defined in Daubechies et al. [8], and 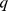 is the relative area of the activation. To achieve the required visual contrasts – small, medium, large and very large boxes, at any desired position – we reorder the two-dimensional samples, never decoupling the realizations of the sources. The final result, while having a similar visual appearance as the experiments of Daubechies et al. [8], retains the joint pdf. This eliminates possible confusion with respect to the influence of the different box parameters on the results of our experiments. We then compute our results using four algorithms: 1) Infomax with the standard sigmoid nonlinearity that assumes a unimodal super-Gaussian source, called Infomax (super); 2) FastICA with the same nonlinearity used in Daubechies et al. [8], which is 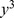; 3) Infomax with a nonlinearity which assumes a sub-Gaussian source, called Infomax (sub); and 4) ICA-EBM (ICA by entropy bound minimization), a much more flexible ICA algorithm [12] able to deal with both super- and sub-Gaussian sources.

Results are averaged over 100 source realizations (each using a different random full-rank mixing matrix 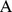) and 10 ICA runs (see Table 1). We also report two performance metrics, first, using the metric chosen in Daubechies et al. [8] 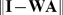, which is *not* invariant to the scaling and permutation ambiguities inherent to ICA. Hence, we also report the results using the inter-symbol interference (ISI), or normalized Moreau-Amari index [13], which is invariant to the scaling and permutation ambiguities: 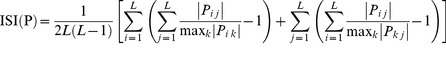
(2)Here, 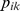 are the elements of the matrix 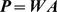, and 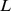 is the number of sources. This performance metric is bounded between 0 and 1 and the lower the ISI value the better the separation performance (the performance metric is zero if and only if the model is identified up to the scaling and permutation ambiguities).

**Table 1:** Source estimates for the four cases indicated in Figure 1.

Box Size & Properties		Results		(good is ISI <0.1)
**A. Small:**		**Algorithm**	**Daubechies**	**Amari (ISI)**
Unimodal, super-Gaussian sources		*FastICA*	**0.0547±0.0150**	**0.0383±0.0107**
**Source** c_1_ (excess) Kurtosis [<0.1 is Gauss-like]:	0.8829	*Infomax (super)*	**0.0331±0.0002**	**0.0228±0.0001**
**Source** c_2_ (excess) Kurtosis [<0.1 is Gauss-like]:	0.8107	*Infomax (sub)*	1.0493±0.0015	0.9499±0.0004
Mutual Information Between Sources c_1_ & c_2_:	0.0920	*ICA-EBM*	**0.0554±0.0066**	**0.0388±0.0047**
***Summary*** *: FastICA, Infomax (super), and ICA-EBM perform well*				
**B. Medium:**		**Algorithm**	**Daubechies**	**Amari (ISI)**
Bimodal, close-to-Gaussian sources		*FastICA*	0.2068±0.0662	0.1464±0.0513
**Source** c_1_ (excess) Kurtosis [<0.1 is Gauss-like]:	0.2564	*Infomax (super)*	0.8722±0.0651	0.7434±0.0600
**Source** c_2_ (excess) Kurtosis [<0.1 is Gauss-like]:	0.0879	*Infomax (sub)*	0.1597±0.0058	0.1144±0.0041
Mutual Information Between Sources c_1_ & c_2_:	0.0929	*ICA-EBM*	**0.0693±0.0105**	**0.0488±0.0075**
***Summary*** *: ICA-EBM performs good, Infomax (sub) performs fair*				
**C. Large:**		**Algorithm**	**Daubechies**	**Amari (ISI)**
Bimodal, close-to-Gaussian sources		*FastICA*	0.4081±0.1003	0.3102±0.0823
**Source** c_1_ (excess) Kurtosis [<0.1 is Gauss-like]:	0.0010	*Infomax (super)*	1.0297±0.0009	0.9236±0.0005
**Source** c_2_ (excess) Kurtosis [<0.1 is Gauss-like]:	0.0762	*Infomax (sub)*	**0.0401±0.0004**	**0.0260±0.0004**
Mutual Information Between Sources c_1_ & c_2_:	0.0892	*ICA-EBM*	**0.0145±0.0008**	**0.0094±0.0008**
***Summary:*** * ICA-EBM and Infomax (sub) perform well*				
**D. Very Large (max kurtosis):**		**Algorithm**	**Daubechies**	**Amari (ISI)**
Unimodal, super- Gaussian sources.		*FastICA*	**0.0263±0.0078**	**0.0180±0.0057**
**Source** c_1_ (excess) Kurtosis [<0.1 is Gauss-like]:	5.6432	*Infomax (super)*	**0.0131±0.0003**	**0.0086±0.0002**
**Source** c_2_ (excess) Kurtosis [<0.1 is Gauss-like]:	5.6394	*Infomax (sub)*	1.0711±0.0014	0.9762±0.0009
Mutual Information Between Sources c_1_ & c_2_:	0.0686	*ICA-EBM*	**0.0218±0.0019**	**0.0148±0.0014**
***Summary*** *: FastICA, Infomax (super), and ICA-EBM perform well*				

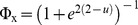
 as in Example 2 of the original paper [8]. The algorithms behave as one would expect if they are selecting for independence. For the bimodal/Gaussian-like cases, ICA-EBM and Infomax (sub) do well, and for the unimodal/maximum kurtosis/low sparsity case Infomax-super, FastICA and ICA-EBM all do extremely well. Numbers in boldface indicate when separation was good.

As expected, the most flexible approach, the ICA-EBM algorithm, performs well (ISI<0.1) in all cases (Table 1). Infomax (sub) performs well to moderately-well for the large and medium boxes, both of which are bimodal and have a kurtosis that is close to that of a Gaussian random variable. Infomax (super) and FastICA perform marginally well or poorly in those cases but perform very well for the cases of very large boxes (maximum kurtosis) and for small boxes. This makes intuitive sense, as high-kurtosis data matches the underlying assumptions of both Infomax (super) and FastICA in that the source distributions are unimodal and strongly super-Gaussian. These results directly contradict the claim in Daubechies et al. [8] that Infomax (super) and FastICA select for sparsity, since the maximum kurtosis case also has the lowest sparsity of the four (again using the first definition of sparsity in Daubechies et al. [8]).

## Sparsity and Sources that Are Mixture of Gaussians

In the sparsity section in Daubechies et al. [8, p.10421, Fig. 8] there are several incorrect statements that are important and require a careful critique. First, Daubechies et al. [8] claims that the sources in the so-called “promotional material for ICA” are Gaussian. We show below that they are in fact highly non-Gaussian. Second, a definition of sparsity different from the one proposed earlier in the paper [8] is used to claim that the sources are sparse. We show that this sparsity does not refer to the sources and in actuality they are not sparse. Finally, we correct several other statements within that section.

### Counter proof to claim of Gaussian sources

**Figure 2:**
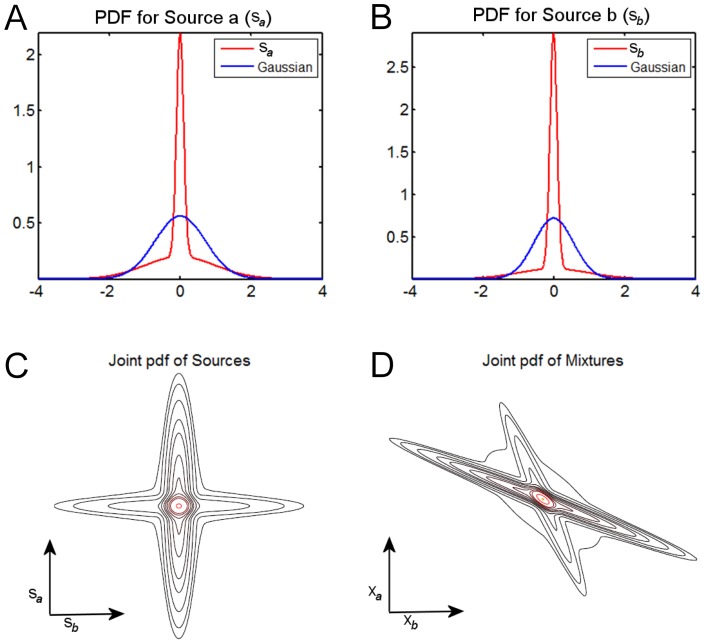
The distribution of sources and mixtures for 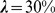 (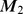). We plot (A–C) the distribution of sources, and (D) the contour plot of mixtures for the case of 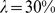 (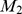). Contrary to the claim made in Daubechies et al., the sources have in fact very peaky and heavy-tailed distributions and are not at all close to a Gaussian distribution. For comparison purposes we also present Gaussian distribution curves (blue, A–B).

To identify the distribution of the sources in this example, it is sufficient to look along the mixing directions 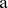 and 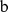. Observations are defined as 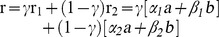. Reordering the terms gives 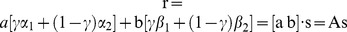, 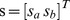, 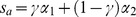, 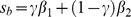. Thus, the sources can be identified as mixture distributions. Their distributions are given as 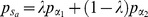 and 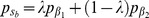, where 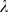 is the parameter of the Bernoulli distribution of which 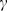 are the realizations. Notice that contrary to what one might expect, a mixture of Gaussian random variables through a Bernoulli random variable 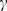 as above, in general does not yield a Gaussian random variable, but rather a random variable whose pdf is a weighted mixture of two independent Gaussian pdfs. Finally, since the distributions 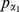, 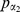, 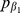, and 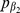 (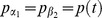) are all Gaussian and no two distributions in a mixture have the same variance – e.g., for the choice in Daubechies et al. [8], 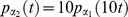, which implies 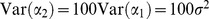 – the resulting distribution must be non-Gaussian whenever 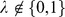. Hence the statement in Daubechies et al. [8] that “Each component has a Gaussian distribution”, is incorrect; the components are in actuality highly non-Gaussian (see Figure 2 (A–B)).

### Critique of the claim of sparse components

In the same section there is a claim that the components (*i.e.* sources) in this example are sparse: “Fig. 8 depicts processes with 2 sparse rather than independent components”. The definition of B-sparsity in this section regards the number of elements (B) in a zero-mean random vector that have variance close to zero. Thus, a 1-sparse 2D random vector means that the variance of one of the two elements is close to zero. However, B-sparsity in this section does not refer to the components at all; rather, it refers to parts of the components together, specifically, the 2D Gaussian vectors 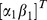 and 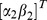, which are 2D, 1-sparse vector processes. In actuality, however, the components 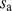 and 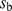 are *not* sparse for the choice of 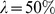 and 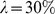 used in 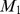 and 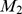, respectively. This is because 
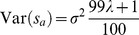
 and 
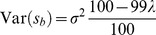
 are far from 0 for the choices of 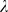 in 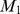 and 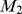, and both are typically 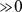 for 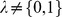. Therefore, it cannot be sparsity that is driving these algorithms towards the solution.

### A few additional clarifications

There are two other sentences in the section on sparsity in Daubechies et al. [8] which require some clarification. First, in the sentence “However, in the example given here, is Gaussian; because ICA methods cannot separate mixtures of independent Gaussian processes, the successful separation of components by Infomax and FastICA underscores again their ability to identify sparse components” 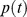 is *not* the distribution of the components 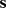. In addition, the statement instills belief that this example has only a single mixing process, when in fact it has two: 1) the mixing of the (Gaussian) 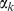
’s and 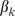
’s through 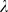, which gives the (non-Gaussian) sources 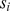, and 2) the mixing of sources 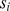 through the mixing matrix 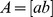. The statement suggests Infomax and FastICA can unmix the Gaussian random variables 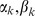 which constitute the mixture distribution of a source (i.e. the two parts of a single source 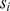) which is clearly incorrect (they unmix the sources 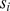, not their subparts). Lastly, the sentence “Infomax or FastICA identify the 2 special directions **a** and **b** correctly as the components” incorrectly labels 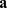 and 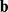 as components, when they actually are the *mixing coefficients* that make up the 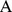 matrix.

## ICA of Sources with Mixture of Gaussians Distribution

The discussion related to the example in Figure 8 of the original paper [8] initially notes that mixtures of independent Gaussian random variables cannot be recovered by ICA, which is true if each source comes from a single Gaussian distribution, and the algorithms are only based on higher-order statistics, as in the case of Infomax and FastICA (i.e., the algorithms do not exploit sample correlation). However, these algorithms (and many others that have been developed and also applied to fMRI data [14]) *can* separate sources whose probability density can be represented via a Gaussian mixture model, as long as the resulting distribution itself is not a Gaussian. The latter is the case in the example presented in Figure 8 of Daubechies et al. [8], which was incorrectly seen as evidence that sparsity was the driving force helping ICA to recover Gaussian sources. We showed that the sparsity mentioned in Daubechies et al. [8] is not related to the sources. Also, this example utilizes a mixture of Gaussians as the sources. With the parameters described in Daubechies et al. [8], the sources are in fact super-Gaussian (i.e. they have positive excess kurtosis, as shown in Table 2). Infomax and FastICA with nonlinearities selected to match a super-Gaussian distribution are expected to successfully separate such sources, as also is the more flexible ICA-EBM algorithm [12]. Conversely, Infomax with a nonlinearity selected to be sensitive to sub-Gaussian sources is expected to exhibit suboptimal performance (see Table 2). This can also be visualized in Figure 2 where we show the sources and the mixtures for the case of 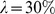 as described in Daubechies et al. [8]. This example again points to the confusion discussed in the Section “The statistical properties of synthetic data in Daubechies et al. [8]” with respect to the definition of the underlying ICA sources, i.e., what is actually being simulated and what is assumed in Daubechies et al. [8].

**Table 2:** Tabulated results for the so-called [8] ICA “promotional material.”

Observed Properties and Results (good is ISI <0.1)		
Property	Source a (s_a_)	Source b (s_b_)
Negentropy:	0.2753	0.3708
(excess) Kurtosis:	3.0630	3.5225
**Algorithm**	**Daubechies**	**Amari (ISI)**
*FastICA*	**0.0154**	**0.0108**
*Infomax (super)*	**0.0076**	**0.0052**
*Infomax (sub)*	1.0758	0.9899
*ICA-EBM*	**0.0059**	**0.0039**

Both Infomax (super) and FastICA do successfully separate (zero ISI indicates perfect separation) the super-Gaussian sources 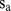 and 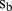. Note the excess kurtosis is more than 3 for both sources. Numbers in boldface indicate when separation was good.

## On the Definition of Sparsity

In coding theory, whether in transmission or in storage of a signal, a trade-off often is necessary between attainable compression rates and signal restoration error. In this context, sparsity is a signal property that allows for high compression rates, while compromising only little in the restoration error. A sparse signal generally consists of 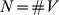 coefficients of which 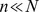 coefficients concentrate all information within the signal. Indeed, under the hypothesis that coding a string of zeroes has little cost in resources with respect to coding whatever floating/integer number, all other 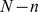 coefficients could be set to zero without significant loss of information but with a substantial gain in compression rate.

**Figure 3:**
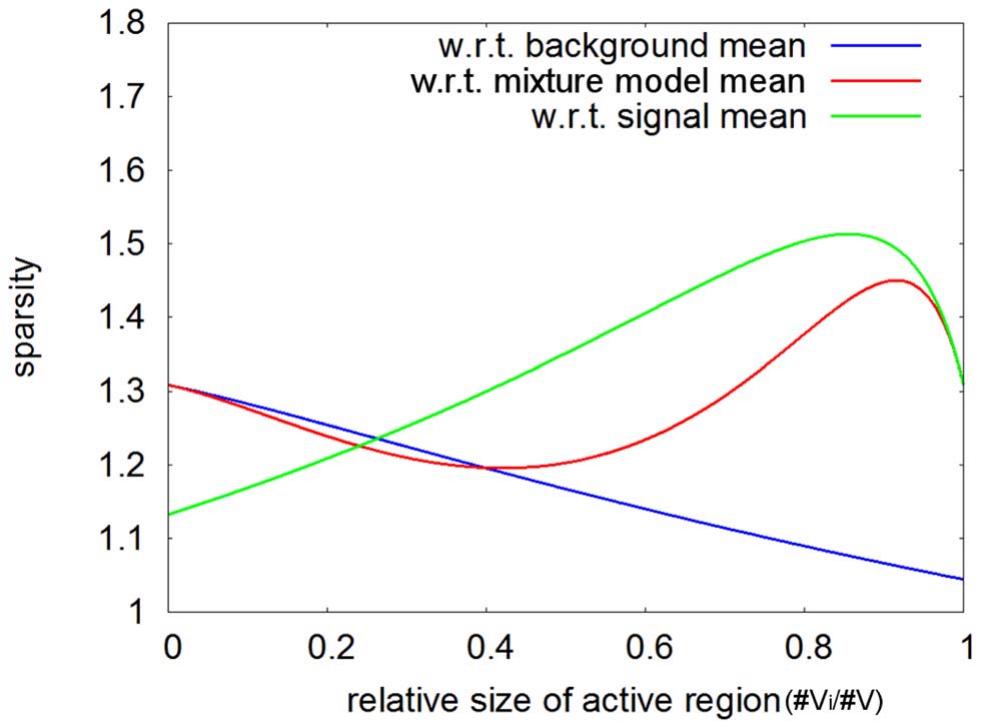
Sparsity measures for three different coordinate system origins (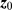). Sparsity as measured with respect to different coordinate system origins (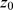), as a function of the relative size of the active region. Remark that for a relative size of zero, only background samples are present and, thus, the mean of the mixture model coincides with the mean of the background (and the two sparsity measures correspond at this point). An analogous observation can be made for a relative size of one, now with respect to the activity (signal samples).

A legitimate question now is what about a signal of which all but 1 coefficient differ from a number, say, 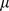. Let that one coefficient equal zero. Is that signal sparse? Under the above definition, the signal would not be considered as sparse, since only a single coefficient could be coded as a zero without introducing a reconstruction error. However, if we would allow for coding a shift by 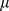, then coding 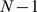 coefficients as zero would result in a reconstruction error 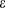 upper bounded by 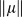 (and we would find 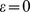 with probability 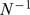). It is clear from this very simple example that it is important to appropriately choose the origin for the coordinate system (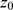) in which one foresees to evaluate the sparseness of the signal. For the model considered in Daubechies et al. [8], we plot the sparsity measure 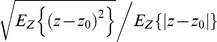 for three different choices of 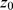. Here, the ordinary sparsity measure (as understood in Daubechies et al. [8]) is taken with respect to 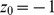, i.e., the mean of the “background distribution”, with sparsity decreasing as the active region size increases (see Figure 3). Note that for fMRI we typically use zero-mean samples when using ICA, thus measuring our sparsity with respect to the mean of the mixture model.

## On the Application of ICA to fMRI

We also note that, contrary to the claims in Daubechies et al. [8], Infomax and FastICA, though the most widely used at the time – due in large part to their availability in fMRI-friendly software packages – were not the only ICA algorithms that had been applied to fMRI analysis with success at the time [15], [16]. This trend has continued and in recent years even more flexible algorithms such as those based on entropy bound minimization (EBM) or full blind source separation (FBSS) have been used increasingly to analyze fMRI data, outperforming both Infomax and FastICA [14], [17], [18]. In general, we would recommend that these and other more recent algorithms preferentially be applied to fMRI, as they are generally more robust to non-super-Gaussian and/or multimodal distributed sources which can occur in real fMRI data, observed in the context of certain artifacts. These algorithms and many others are implemented in the group ICA of fMRI toolbox (GIFT; http://mialab.mrn.org/software/gift). An interesting historical note is that before extended Infomax [19] was introduced, there was confusion as to how ICA of fMRI really worked when it was applied as temporal ICA and early results indeed were not convincing – since time courses are more likely to be sub-Gaussian than super-Gaussian [20], whereas in the spatial ICA case super-Gaussian sources are more common. Another important point regarding the real fMRI experiment mentioned in Daubechies et al. [8] is that each voxel is identified as belonging to only one underlying source (page 10416, left col, third paragraph). Such an approach is perhaps a reflection of the way one might approach an fMRI experiment with a sparsity focus, but in reality, and more in line with the complexity and connectivity of the human brain, each voxel typically has a contribution from multiple components (sources), making this an ideal case for ICA.

## Conclusions

We reviewed the main claim made in Daubechies et al. [8] and its supporting evidence. We revisit the initial experiments and present new evidence showing conclusively that the arguments fall short of supporting the claim that Infomax and FastICA select for sparsity and not for independence. While pointing out that the use of other metrics for fMRI analysis such as sparsity – besides independence, which is widely used – is a reasonable goal, the claims that are used to justify this desire are misleading at best and in some cases are simply incorrect. In summary, we show that ICA algorithms, including FastICA and Infomax, are indeed doing what they were designed to do, maximize independence.
