# Correction: Independent Component Analysis for Brain fMRI Does Indeed Select for Maximal Independence

**DOI:** 10.1371/annotation/52c7b854-2d52-4b49-9f9f-6560830f9428

**Published:** 2013-10-24

**Authors:** Vince D. Calhoun, Vamsi K. Potluru, Ronald Phlypo, Rogers F. Silva, Barak A. Pearlmutter, Arvind Caprihan, Sergey M. Plis, Tülay Adalı

This article was republished on October 23, 2013 because of missing equations. Please download this article again to view the equations. 

